# Molecular Docking and ADME-TOX Profiling of *Moringa oleifera* Constituents against SARS-CoV-2

**DOI:** 10.3390/arm91060035

**Published:** 2023-10-27

**Authors:** Hellen Cris Araújo Souza, Maycon Douglas Araújo Souza, Cássio Silva Sousa, Edilanne Katrine Amparo Viana, Sabrina Kelly Silva Alves, Alex Oliveira Marques, Arthur Serejo Neves Ribeiro, Vanessa de Sousa do Vale, Muhammad Torequl Islam, João Antônio Leal de Miranda, Marcelo da Costa Mota, Jefferson Almeida Rocha

**Affiliations:** 1 Medicinal Chemistry and Biotechnology Research Group—QUIMEBIO, São Bernardo Science Center, Federal University of Maranhão UFMA, São Bernardo 65080-805, MA, Brazil; hellen.cris@discente.ufma.br (H.C.A.S.); maycon.araujo@discente.ufma.br (M.D.A.S.); cassio.silva@discente.ufma.br (C.S.S.); edilanne.katrine@discente.ufma.br (E.K.A.V.); sabrina.kelly@discente.ufma.br (S.K.S.A.); ao.marques@discente.ufma.br (A.O.M.); ribeiro.arthur@ufma.br (A.S.N.R.); vanessa.vale@ufma.br (V.d.S.d.V.); marcelo.mota@ufpi.edu.br (M.d.C.M.); ja.rocha@ufma.br (J.A.R.); 2 Department of Pharmacy, Bangabandhu Sheikh Mujibur Rahman Science and Technology University, Gopalganj 8100, Bangladesh; dmt.islam@bsmrstu.edu.bd; 3 Department of Medicine, Senador Helvidio Nunes de Barros Center, Federal University of Piauí (UFPI), Picos 64607-670, PI, Brazil

**Keywords:** antiviral activity, SARS-CoV-2, *Moringa oleifera*, computational medicinal chemistry

## Abstract

**Highlights:**

**What are the main findings?**

- Bioactive compounds of *Moringa oleifera* exhibited activity against SARS-CoV-2.
- Computational approaches to studying the antiviral activity of natural compounds against SARS-CoV-2 might be a time- and money-saving option in the drug discovery and development process.

**What is the implication of the main finding?**

- The antiviral potential of *Moringa oleifera* against SARS-CoV-2 may contribute to an advanced level of pharmaceutical research.
- Advanced computational methods can be used to search for novel anti-SARS-CoV-2 agents from natural products.

**Abstract:**

The SARS-CoV-2 (severe acute respiratory syndrome coronavirus 2019) etiological agent, which has a high contagiousness and is to blame for the outbreak of acute viral pneumonia, is the cause of the respiratory disease COVID-19. The use of natural products grew as an alternative treatment for various diseases due to the abundance of organic molecules with pharmacological properties. Many pharmaceutical studies have focused on investigating compounds with therapeutic potential. Therefore, this study aimed to identify potential antiviral compounds from a popular medicinal plant called *Moringa oleifera* Lam. against the spike, M^pro^, ACE2, and RBD targets of SARS-CoV-2. For this, we use molecular docking to identify the molecules with the greatest affinity for the targets through the orientation of the ligand with the receptor in complex. For the best results, ADME-TOX predictions were performed to evaluate the pharmacokinetic properties of the compounds using the online tool pkCSM. The results demonstrate that among the 61 molecules of *M. oleifera*, 22 molecules showed promising inhibition results, where the compound ellagic acid showed significant molecular affinity (−9.3 kcal.mol^−1^) in interaction with the spike protein. These results highlight the relevance of investigating natural compounds from *M. oleifera* as potential antivirals against SARS-CoV-2; however, additional studies are needed to confirm the antiviral activity of the compounds.

## 1. Introduction

In December 2019, a new respiratory disease called Noble Coronavirus Disease 2019 (COVID-19) was identified in Wuhan, China. The etiologic agent involved is the new severe acute respiratory syndrome coronavirus 2 (SARS-CoV-2) [1]. It is a positive-sense RNA beta-coronavirus [2] that doesn’t have any segments and has a high rate of spreading. It is what caused the acute viral pneumonia outbreak.

In recent years, the use of medicinal plants in the treatment of various diseases has increased because this approach has gained credibility as a result of important pharmaceutical research [3,4]. Among these plants, we highlight the genus Moringa, the only representative of the Moringaceae family, which comprises fourteen species widely distributed in the tropical regions of the planet [5,6]. Among the species described for the genus is *Moringa oleifera* Lam. In Brazil, it was introduced as an ornamental plant around 1950 [7], and since then, it has been widely cultivated due to its high nutritional value, especially the leaves, which are rich in carotene, ascorbic acid, and iron [8,9].

All parts of *M. oleifera* are traditionally used for different purposes, but the leaves are the most commonly used [10]. These can be consumed directly, raw and dried, or in the form of an aqueous infusion to treat various ailments, including malaria, typhoid fever, parasites, arthritis, swelling, skin diseases, hypertension, and diabetes. In addition to being used to induce lactation and improve the immune system, *M. oleifera* is characterized by having a high concentration of proteins, vitamins, minerals, β-carotene, and secondary metabolites with antioxidant properties, including glucosinolates, flavonoids, and phenolic acids, which have effects against chronic diseases [11,12]. This plant is easily found in tropical and subtropical regions; therefore, these classes of people are very popular with it [13].

The computer techniques used in bioinformatics help procedures in this field because they save time and money and speed up the process of obtaining results from experiments in vitro and in vivo. This is because they make it easier to organize data and help choose targets or hypotheses to be tested on the bench [14,15,16]. These approaches are essential for identifying promising compounds with pharmacological potential for the development of new drugs. In this way, finding a promising treatment becomes a top priority, and it is important to use computational methods to quickly find compounds that have a molecular affinity for the proteases of the SARS-CoV-2.

Several studies were done to scientifically prove that *Moringa* can be used to treat these illnesses because it is thought to have healing properties [17,18,19]. Different parts of the tree, like the root, bark, gum, leaves, fruits (pods), and flowers, have different health benefits. For example, they can help with allergies, fight cancer, lower blood sugar, fight fungal growth, protect the liver, and boost the immune system [20].

Muratov et al., (2021) [21] said that we need to carefully look at computational approaches in order to find effective treatments for SARS-CoV-2. The pandemic motivated global efforts to identify therapeutic approaches, with an emphasis on computational research. Effective integration of these tools with experimentation is crucial for validating results and developing antiviral therapies. This underscores the need for a multidisciplinary approach where computational research identifies drug candidates and clinical trials validate these findings.

Compounds derived from natural sources have significant therapeutic value and make up more than half of the drugs approved by the FDA [22]. Natural products represent a valuable source of bioactive molecules for drug screening. To date several studies report that natural products have anti-SARS-CoV-2 effects. The virtual screening of natural products using the molecular docking method plays a crucial role in evaluating the inhibitory activity of these molecules against SARS-CoV-2. However, these findings should be validated through in vitro studies [23].

Power et al., (2022) [24] screened ten natural compounds through in silico studies and found favorable ADMET profiles for the in vitro evaluation of their activity against SARS-CoV-2. During the in vitro analysis, four compounds were active against SARS-CoV-2, suggesting the application of in silico studies in in vitro evaluation in the drug discovery field.

The compounds present in the plant are promising for research aimed at finding substances that will have good results against the essential proteins of SARS-CoV-2. With the help of tools in the molecular docking method, it is possible to figure out the best way for the compound and the target protein to connect at the molecular level [25]. This function lets you figure out how the compound acts in the active site of a pathogen’s key protein and see the molecular interactions between the compound and the protein [26]. This tool also allows virtual drug screening and the characterization of molecular structures [27]. Thus, this study aims to identify compounds with inhibitory potential based on the mechanisms of action in complex with SARS-CoV-2 targets and to make predictions of the absorption, distribution, metabolization, excretion, and toxicity (ADME-TOX) of *M. oleifera* compounds.

## 2. Materials and Methods

### 2.1. Selection of Binders

A total of 61 chemical compounds from the *M. oleifera* species were selected, covering classes such as flavonoids, alkaloids, terpenoids, coumarins, and saponins, among others, in national and international scientific databases such as Scientific Electronic Library Online (Scielo), National Center for Biotechnology Information (PubMed), Elsevier Group (Scopus and Science Direct), and Google Scholar. The search was conducted using the keyword “*Moringa oleifera*” in combination with “chemical constituents” or “phytochemicals”. The corresponding chemical structures were acquired through the Pubchem platform (http://pubchem.ncbi.nlm.nih.gov/, accessed on 3 September 2022).

### 2.2. Molecular Docking

The 3D structures of three coronavirus targets were obtained from the Protein Data Bank (PDB) database (http://www.rcsb.org/, accessed on 10 October 2022) [28], with the respective codes: spike protein (PDB ID: 6VXX), angiotensin converting enzyme—ACE2 (PDB ID: 1R42), main protein Mpro (PDB ID: 6LU7), while the Receptor5 (RBD–spike/ACE2 interaction site) was designed by Barros et al., (2020) [29]. For molecular affinity, they were prepared by removing all water molecules and other groups, such as ions, using Chimera V software. 13.1 [30]. Afterwards, polar hydrogen atoms were added, the Gasteiger partial charges were calculated, and the non-polar hydrogens were merged in both parts (protein and ligand) using the Autodock Tools (ADT) program version 1.5.6. Subsequently, the docking was carried out using the program AutoDock Vina [31]. The grid box size was set to 30 Å for each axis. The grid boxes were centered on the coordinates of the atoms of the residues located in the active site region and interface region, namely: Gly548(A) (6VXX), His374 (A) (1R42), Gly143(A) (6LU7), and Phe32(B) (Receptor 5) (Table 1). The number of modes was set to 50, and the exhaustiveness was set to 24. With the LIGPLOT program, 2-D diagrams of protein–ligand complexes were made from the PDB file, which was the standard input. Pictures were made to show where the hydrogen and hydrophobic bonds of the compounds interact with the amino acids of the viral proteins [32]. The analyses were concentrated on the lower energy complexes of the socket conformation. The lowest energy conformations, combined with visual inspection, were chosen for a more detailed analysis [16].

In this study, the PDB structures of three proteins were selected as main targets for virtual screening of compounds aimed at discovering potential antiviral agents against SARS-CoV-2: the main protease (Mpro) (PDB ID: 6LU7), the spike glycoprotein (S) (PDB ID: 6VXX), and the functional receptor ACE2 (PDB ID: 1R42). This selection was based on their recognized importance in the virus life cycle and infection mechanism.

M^pro^ was selected as a target due to its essential function in viral replication. The main protease (M^pro^) is responsible for the cleavage of the viral polyprotein into independent functional proteins, which is necessary for the replication of the SARS-CoV-2 virus [33]. Its inhibition can effectively halt the viral replication process, making it a promising target for antiviral therapies. Previous studies highlighted the relevance of Mpro as a therapeutic target, and the PDB structure of M^pro^ (PDB ID: 6LU7) was used as the basis for our molecular docking simulations [34,35].

The spike (S) glycoprotein was also chosen as a target due to its fundamental role in virus entry into host cells. The spike protein’s receptor-binding domain (RBD) connects with the host cell’s functional ACE2 receptor, which helps the virus infect the cell [36]. Existing research indicates that the spike is a relevant target for the search for inhibitors, as interrupting this early stage of the viral cycle is an effective strategy for controlling infection. The PDB structure of spike (PDB ID: 6VXX) was used as a reference for our molecular docking analyses [37,38].

The functional ACE2 receptor was also considered an important target, as it plays a central role in the binding and internalization of SARS-CoV-2 into host cells [39]. Inhibition of ACE2 can block the interaction between the viral spike and the host cell, preventing infection. Studies on inhibitors against SARS-CoV-2 included ACE2 as a molecular target, aiming to interrupt the COVID-19 infection process [37,40]. Therefore, these proteins were chosen as research targets because they play a key role in the life cycle of viruses and could be used as targets for antiviral drugs.

### 2.3. ADME-TOX Prediction

The prediction of pharmaceutical parameters was performed using the pkCSM pharmacokinetics software (https://biosig.lab.uq.edu.au/pkcsm/, accessed on 10 February 2023), available free of charge [41]. The in silico methodology used with the molecules ellagic acid, rutin, myricetin, quercetin, luteolin, isoquercetrin, isorhamnetin, kaempferol, chlorogenic acid, lutein, catechin, apigenin, glucomoringin, epicatechin, brassicasterol, stigmasterol, and ergosterol included physical-chemical parameters, pharmacokinetic profile (ADME), and toxicity. Parameters include absorption (water solubility, Caco-2 permeability, human intestinal absorption, skin permeability, and P-glycoprotein I and II inhibitor), distribution (steady-state volume of distribution (VDss) and blood–brain barrier permeability), metabolism (CYP2D6 and CYP3A4 substrate, CYP1A2, CYP2C19, CYP2C9, CYP2D6, and CYP3A4 substrate), excretion (OCTC renal substrate), and toxicity (AMES toxicity, maximum tolerated dose, hERG I and II inhibitor, acute oral toxicity in rats (LD_50_), chronic oral toxicity in rats (LOAEL), hepatotoxicity, and skin sensitization) [16].

## 3. Results

### 3.1. Molecular Docking

*M. oleifera* plants were tested through the molecular docking process with four receptors that are essential in the process of viral infection and replication. Among the receptors, two proteins are from the virus: the spike glycoprotein and the main protein (M^pro^). In addition to these, ACE2 (angiotensin converting enzyme) and receptor 5 (RBD) were also used.

A total of 244 dockings were performed with the 61 compounds and the four receptors. The results show an energy variation from −3.4 to −9.3 kcal/mol. The lower the binding energy between the compound and the receptor, the better the complex interaction (Table 2).

**Figure 1:**
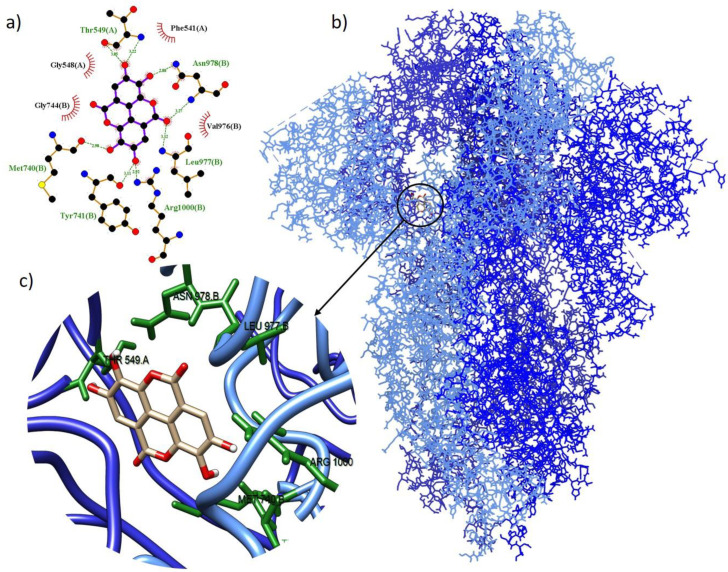
Molecular coupling of the ellagic acid ligand (red) with the spike protein results in a binding free energy of −9.3 kcal/mol (**a**) 2D scheme showing hydrogen bonds (green) and hydrophobic interactions (black); (**b**) site of interaction of the protein-ligand complex; and (**c**) 3D conformation of the binding site of ellagic acid with the spike (S) protein.

To select the best energy parameters, interactions smaller than −7.9 kcal/mol were considered, thus obtaining the 22 interactions described in Table 3. The lowest binding energy (−9.3 kcal/mol) was obtained through the interaction of the ellagic acid compound with the spike protein of SARS-CoV-2 (Figure 1). The complex formed six hydrogen bonds with amino acids: Asn978, Leu977, Arg1000, Tyr741, Met740, and Thr549, and four hydrophobic interactions: Phe541, Val976, Gly744, and Gly548. 

**Figure 2:**
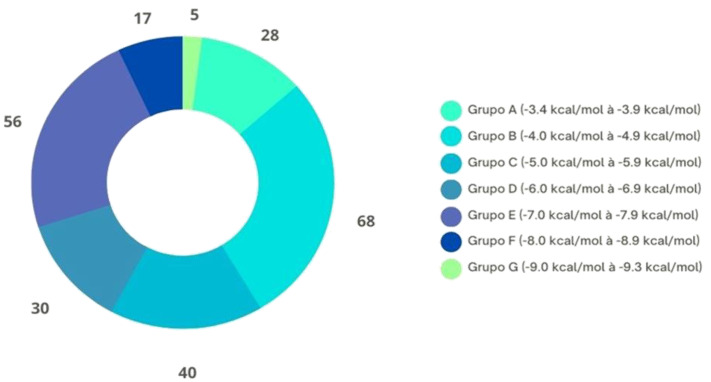
Total number of results presented in terms of binding energy (kcal/mol), organized by categories.

Figure 2 shows all docking performed in this study, with binding energies ranging from −3.4 to −9.3 kcal/mol. The group that obtained the most interactions was group B, which presented energies from −4.0 to −4.9 kcal/mol. Two groups, F and G, had the lowest number of molecular interactions, 22 in total, with energies ranging from −8.0 to −8.9 kcal/mol and from −9.0 to −9.3 kcal/mol, respectively. However, these were the results of the molecular interaction of the ligands with the targets, which was considered more satisfactory in this study (Table 3).

**Figure 3:**
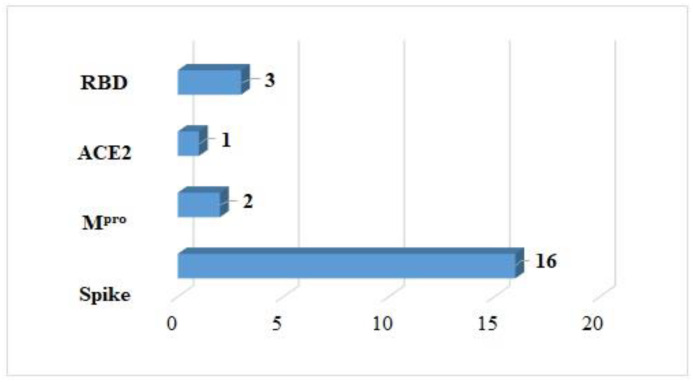
SARS-CoV-2 proteins that demonstrate high levels of interaction with *Moringa oleifera* compounds.

**Figure 4:**
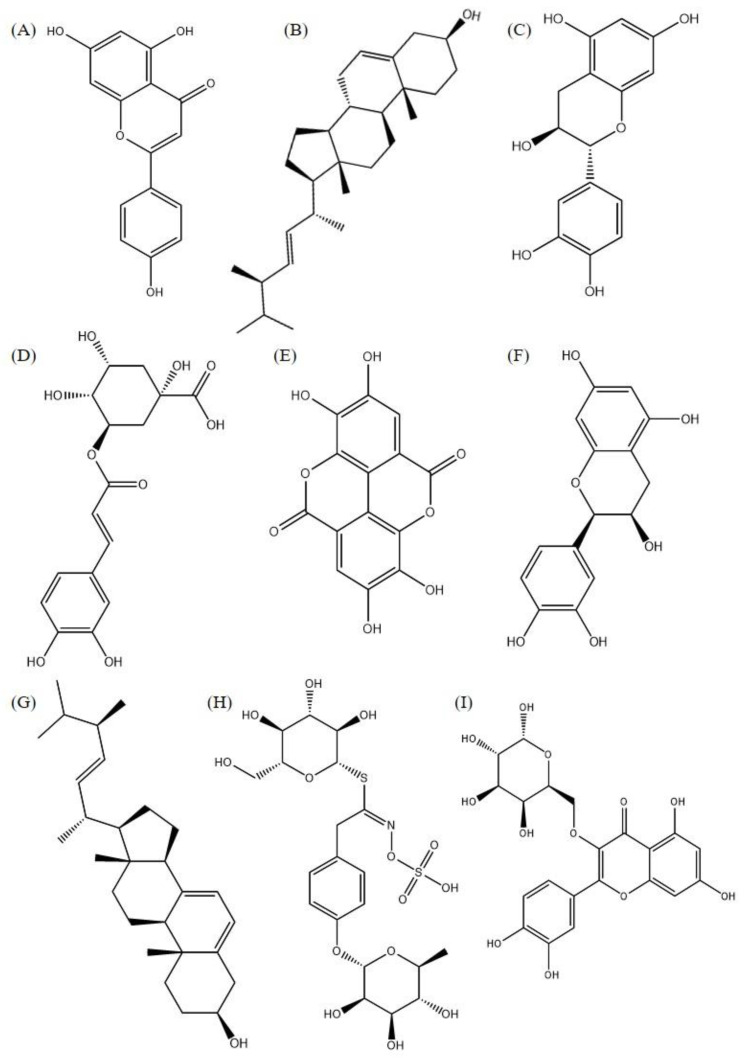

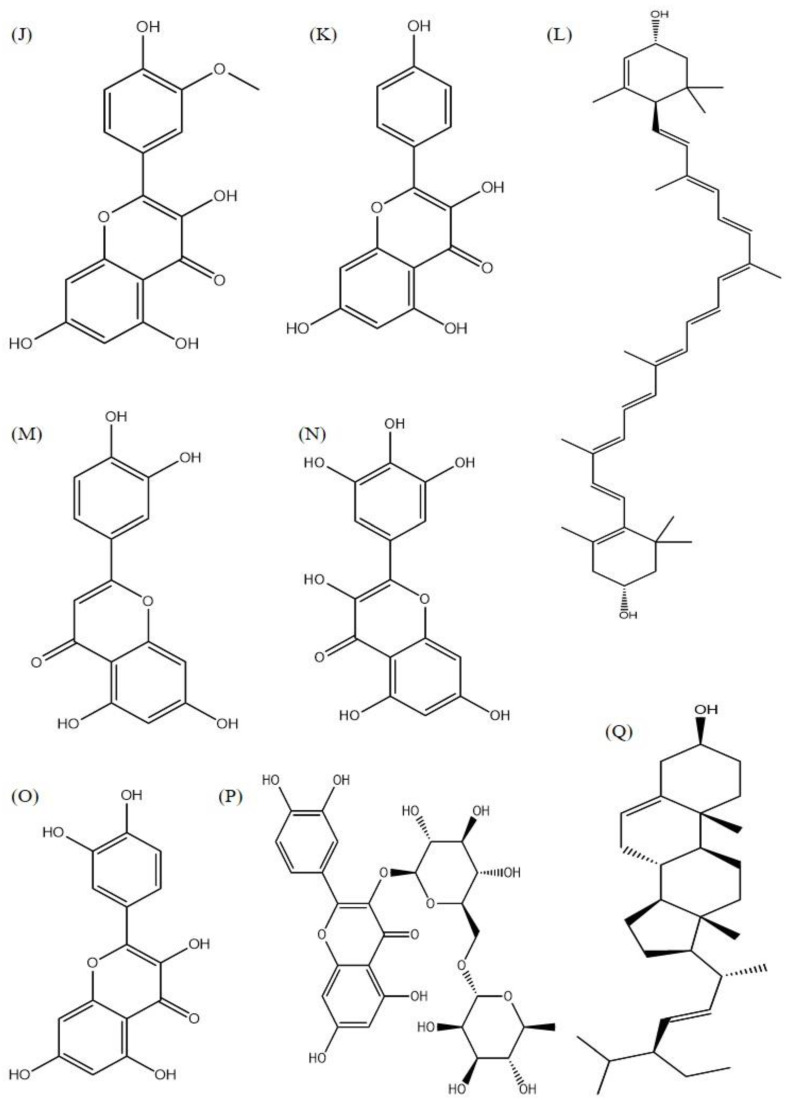
Two-dimensional chemical structure (2D) of the molecules that presented the best results in molecular interaction ((**A**) (Apigenin); (**B**) (Brassicasterol); (**C**) (Catechin); (**D**) (Chlorogenic acid); (**E**) (Ellagic acid); (**F**) (Epicatechin); (**G**) (Ergosterol); (**H**) (Glucomoringin); (**I**) (Isoquercetrin); (**J**) (Isorhamnetin); (**K**) (Kaempferol); (**L**) (Lutein); (**M**) (Luteolin); (**N**) (Miricetin); (**O**) (Quercetin); (**P**) (Rutin); and (**Q**) (Stigmasterol)).

The best molecular interactions with the receptors shown in Figure 3 come from 14 ligands (Figure 4). Some of these ligands had good interactions with more than one protein. It was the spike protein that stood out as the receptor with the most effective complex interactions. It made connections with 16 different compounds. This protein is important for a virus to get into a cell because it interacts with ACE2. Two ligands worked well for the anchoring process with Mpro, which is in charge of virus replication. However, only one compound interacted with ACE2, which is the cell receptor that lets the SARS-CoV-2 virus into the body. Finally, three coupling results with RBD were obtained.

**Figure 5:**
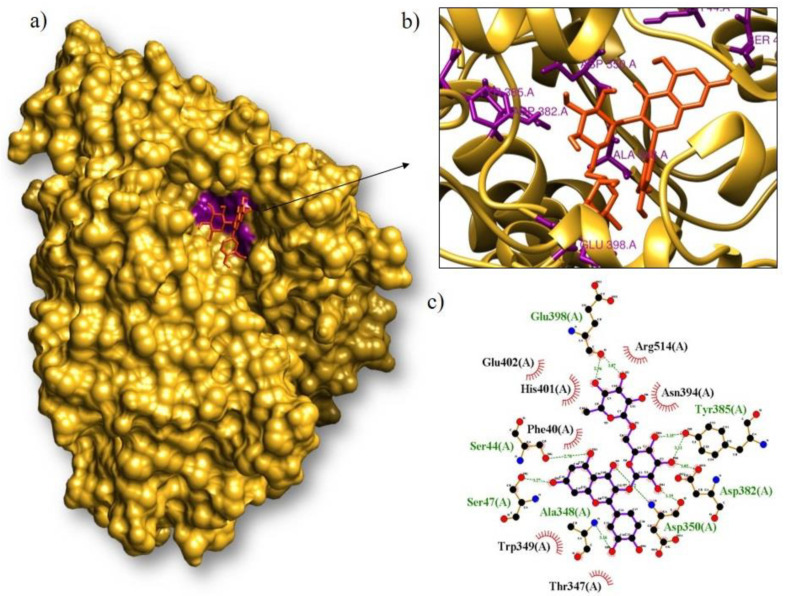
Molecular coupling of the ligand rutin (orange) with the ACE2 protein results in a binding free energy of −8.2 kcal/mol ((**a**) site of interaction of the protein-ligand complex; (**b**) 3D conformation of the binding site of rutin with the ACE2 protein; and (**c**) 2D scheme showing hydrogen bonds (green) and hydrophobic interactions (black)).

Rutin, isoquercitrin, and lutein were the molecules that interacted best with proteins ACE2, M^pro^, and RBD during the molecular docking process. Figure 5 shows that the compound rutin formed the complex with the cellular protein ACE2 that had the lowest interaction energy. It had an interaction energy equal to binding of −8.2 kcal/mol. Seven amino acids (Glu398, Tyr385, Asp382, Asp350, Ala348, Ser47, and Ser44) and seven amino acids (Arg514, Asn394, Thr347, Trp349, Phe40, His401, and Glu402) had hydrogen bonds with it.

**Figure 6:**
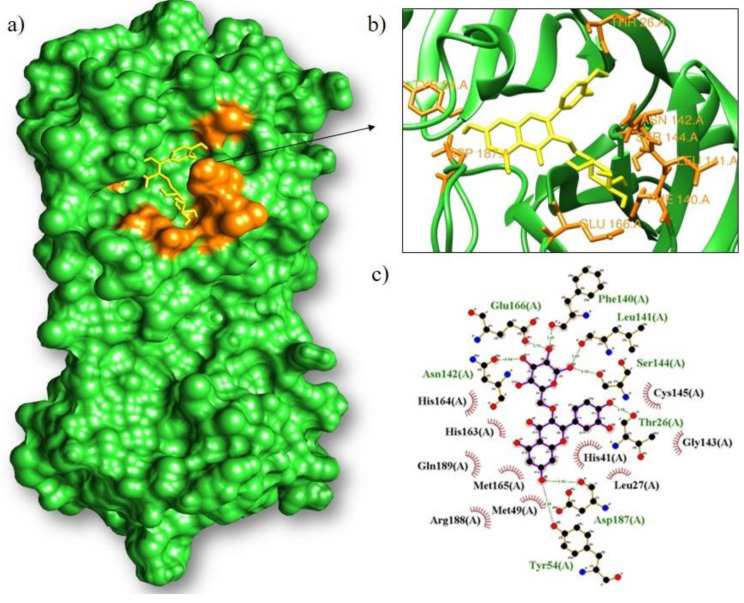
Molecular coupling of the isoquercitrin ligand (yellow) with the M^pro^ results in a binding free energy of −8.9 kcal/mol ((**a**) site of interaction of the protein-ligand complex; (**b**) 3D conformation of the binding site of isoquercetrin with the M^pro^ protein; and (**c**) 2D scheme showing hydrogen bonds (green) and hydrophobic interactions (black)).

**Figure 7:**
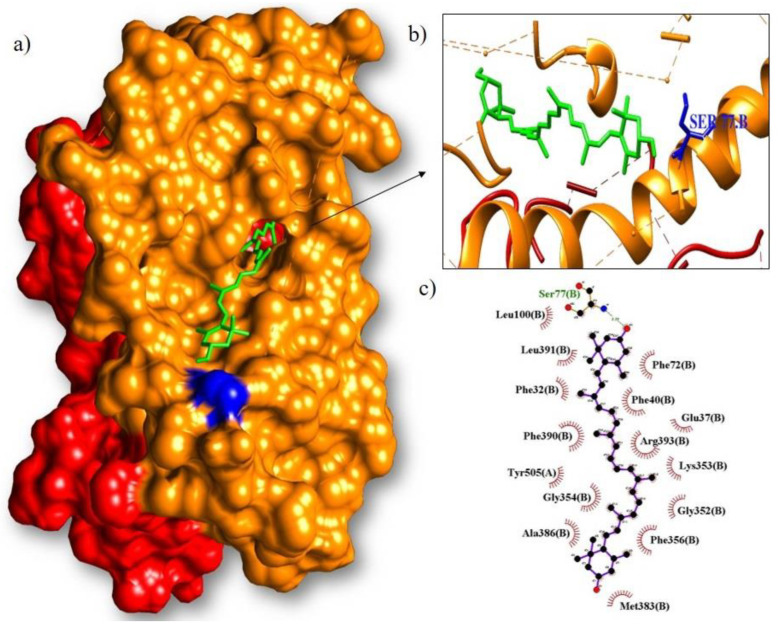
Molecular coupling of the lutein ligand (green) with the RBD results in a binding free energy of −8.7 kcal/mol ((**a**) site of interaction of the protein-ligand complex; (**b**) 3D conformation of the lutein binding site with the spike/ACE2 complex and (**c**) 2D scheme showing hydrogen bonds (green) and hydrophobic interactions (black)).

Isoquercitrin had the best interaction with the main protease (M^pro^), with a binding energy of −8.9 kcal/mol (Figure 6). It formed hydrogen bonds with eight amino acids (Phe140, Leu141, Ser144, Thr26, Asp187, Ty r54, Asn142, and Glu166) and hydrophobic bonds with ten amino acids (Cys145, Gly143, Leu27, His41, Met49, Arg188, Met165, Gln189, His163, and His164). On the other hand, lutein showed a better binding capacity with RBD (−8.7 kcal/mol) (Figure 7). It interacted with an amino acid (Ser77) by hydrogen bonding and by hydrophobic interaction with fifteen amino acids (Phe72, Phe40, Glu37, Arg393, Lys353, Gly352, Phe356, Met383, Ala386, Gly354, Tyr505, Phe390, Phe32, Leu391, and Leu100).

Several studies have been done on the pharmacological properties of different natural compounds and how they can be used to treat and prevent different diseases. These are important for the study and development of promising therapeutic candidates for various diseases. In this study, the molecular docking method was used. This method shows how the complex interacts with the macromolecule and how the compound blocks the action of the macromolecule. Through this method, the ligands ellagic acid, rutin, myricetin, luteolin, and quercetin, as well as other bioactive compounds that, after analyzing the in-silica results (Table 3), showed satisfactory binding energies in their interactions with the receptors, were shown to have inhibitory effects on the molecular targets spike, M^pro^, ACE2, and RBD, thus presenting antiviral effects against SARS-CoV-2. This is a preliminary study regarding activity against the virus. Further tests and in-depth studies about these compounds are needed in order to obtain more knowledge about their properties as promising potential therapeutic candidates in the treatment of this disease.

In Brazil, the National Health Surveillance Agency (ANVISA), the government agency responsible for controlling, monitoring, inspecting, and regulating the production, distribution, and marketing of medicines in the country, granted approval for the emergency use of six medicines intended for the treatment of COVID-19 as of June 2021. Among these drugs, remdesivir, paxlovid (nirmatrelvir + ritonavir), molnupiravir, and baricitinib stand out, being also recommended by the International Solidarity Initiative, led by the World Health Organization (WHO) [50,51]. The results obtained from the interaction of these drugs with SARS-CoV-2 receptors are described in Table 4. Showing that none of the drugs showed results lower than −8.0 kcal/mol.

We can observe that the drugs remdesivir (−7.9 kcal/mol) and paxlovid (−7.6 kcal/mol) had a greater interaction with M^pro^. The bioactive compounds from the *Moringa* species were looked at in this study. Isoquercitrin and rutin interacted better with M^pro^ than the two drugs, with −8.9 and −8.8 kcal/mol, respectively. In addition to these, kaempferol (−7.8 kcal/mol), glucomoringin (−7.9 kcal/mol), and apigenin (−7.7 kcal/mol) also showed better results than paxlovid.

Two other drugs, molnupiravir and baricitinib, showed greater molecular affinity with the spike protein, with binding energies of −7.9 and −8.0 kcal/mol, respectively. However, in this study, there were compounds that showed better interactions with the same protein, namely: ellagic acid (−9.3 kcal/mol), rutin (−9.1 kcal/mol), myricetin (−9.1 kcal/mol), quercetin (−9.0 kcal/mol), luteolin (−9.0 kcal/mol), isorhamnetin (−8.8 kcal/mol), kaempferol (−8.7 kcal/mol), chlorogenic acid (−8.7 kcal/mol), isoquercetrin (−8.6 kcal/mol), catechin (−8.6 kcal/mol), apigenin (−8.6 kcal/mol), glucomoringin (−8.5 kcal/mol), epicatechin (−8.4 kcal/mol), and the compounds brassicasterol, stigmasterol, and ergosterol that showed binding energy (−8.0 kcal/mol) greater than that of molnupiravir and equal to baricitinib.

### 3.2. ADME-TOX Prediction

A computer method called pharmacokinetic prediction in silico is used to look into the ADMET properties of naturally occurring organic molecules that make living things work. This evaluates the absorption, distribution, metabolism, excretion, and toxicity of the molecules. The absorption prediction parameters of the compounds that obtained satisfactory binding energies with the SARS-CoV-2 targets are described in Table 5.

By looking at the molecules’ characteristic absorption parameter, it was shown that most of the compounds tested could dissolve in water within the range of −1 to −5 (mol/L). This means that the compounds have a good hydrophilic capacity. Except lutein, brassicasterol, stigmasterol, and ergosterol, which presented values below −6 (mol/L). Regarding skin permeability, compounds with values above −2.5 cm/h are considered to have low skin permeability. All compounds showed values in the range of −2.799 cm/h to −2.735 cm/h, which indicates that all molecules are considered permeable to the skin. 

Apigenin had a permeability value of 1076 cm/s, brassicasterol had a value of 1209 cm/s, stigmasterol and ergosterol each had a value of 1.21 cm/s, and lutein had a value of 1284 cm/s. Such compounds had values greater than 0.90. The other compounds did not present satisfactory results in this regard. Of the analyzed compounds, only three inhibited P-gp I (brassicasterol, stigmasterol, and ergosterol), while four compounds inhibited P-gp II (lutein, brassicasterol, stigmasterol, and ergosterol). Importantly, the same compounds inhibited P-gp I and II.

Analysis of human intestinal absorption (AIH) is one of the most important ways to judge new drug candidates. The vast majority of molecules analyzed demonstrated an intestinal absorption range between 72.5 and 94.7%, which indicates effective absorption. Isorhamnetin, kaempferol, lutein, catechin, apigenin, epicatechin, brassicasterol, stigmasterol, and ergosterol are some of the molecules that are in this group. That being said, it was seen that compounds like glucomoringin, isoquercitrin, rutin, and myricetin did not absorb well in the intestines.

The VDss (steady-state volume of distribution) is the theoretical volume at which a drug dose needs to be uniformly distributed to result in the same concentration as in blood plasma. The VDss is considered low for values below 0.71 L/kg and high for values above 2.81 L/kg. All compounds showed low VDss; that is, they are all more likely to be distributed in plasma than in tissues. 

Regarding the potential for penetration of the blood–brain barrier (BBB), most compounds have a low potential to cross it. The compounds that were able to cross it showed values with logBB > 0.3; these were brassicasterol, stigmasterol, and ergosterol. Data on how cytochrome P450 proteins (CYP) interact show that some molecules block CYP1A2, CYP2C19, and CYP2C9 from breaking down other drugs in the body. In the study at hand, it was found that apigenin blocks three enzymes (CYP1A2, CYP2C19, and CYP2C9), while ellagic acid, myricetin, quercetin, luteolin, and isorhamnetin only block CYP1A2. None of the evaluated molecules showed inhibition of the CYP2D6 and CYP3A4 proteins or the CYP2D6 substrate. On the other hand, the compounds lutein, brassicasterol, stigmaterol, and ergosterol inhibited the CYP3A4 substrate (Table 6).

Checking if a substance is likely to be carried by OCT2 gives useful details about how it leaves the body, which is necessary to know if a substance is a substrate for OCT2 or not. The results of the analysis demonstrate that none of the evaluated molecules are substrates for OCT2. This information is relevant to understanding the pharmacokinetic behavior of the test compounds, especially in the context of renal elimination.

In order to evaluate the mutagenic potential of the selected compounds, we performed the Ames toxicity test. A positive result indicates that the compound is mutagenic, which may suggest its carcinogen potential. Of the 17 compounds analyzed, 11 (ellagic acid, luteolin, isorhamnetin, kaempferol, chlorogenic acid, lutein, apigenin, glucomoringin, brassicasterol, stigmasterol, and ergosterol) were negative for the Ames test; that is, they are not mutagenic. On the other hand, rutin, myricetin, quercetin, isoquercitrin, catechin, and epicatechin showed positive results, which means they are mutagenic and can cause cancer (Table 7).

Lethal concentration values (LC_50_) represent the concentration of a molecule required to cause 50% of the flathead minnows to die. According to this study, it can be demonstrated that isoquercitrine might be most harmless, while catechin and epicatechin might be harmful, since the higher the lethal dose, the lower the degree of toxicity. Chronic oral toxicity in rats (LOAEL) is analyzed in the same way. Defined as the lowest dosage for observation of adverse effects, it had its most significant result for the compound rutin, which can be ingested in substantial amounts without causing chronic diseases.

The recommended maximum tolerated dose (MRTD) provides an estimate of the threshold toxic dose of chemicals in humans. For the analysis, an MRTD lower than or equal to 0.477 log (mg/kg/day) is considered low. The compounds lutein, glucomoringin, brassicasterol, stigmasterol, and ergosterol showed low values; therefore, they have low toxicity, while the other analyzed compounds showed high values.

The analysis demonstrates whether a given compound is likely to be a hERG I/II inhibitor. So, the analysis showed that no compound can stop hERG I from working. However, seven compounds were found to stop hERG II from working. These compounds are rutin, isoquercitrin, lutein, apigenin, brassicasterol, stigmasterol, and ergosterol.

## 4. Discussion

With the advent of COVID-19, there was a need to identify new substances with antiviral properties against SARS-CoV-2. Faced with this urgency, several clinical studies explored the concept of repositioning existing drugs as an agile approach to developing a new, effective therapeutic model. This scenario provided an opportunity to investigate alternative treatments, with an emphasis on the potential use of medicinal plants [52].

The study’s main goal is to find compounds from M. oleifera that might be good at stopping the activity of SARS-CoV-2 targets, like the spike protein, Mpro, ACE2, and RBD, which would then lower or stop the virus from replicating. It is notable that previous studies searched for effective inhibitors of natural origin from plants with pharmacological activity, which are known in the literature for their use in the treatment and cure of various diseases.

In this study, molecular docking was performed to investigate the antiviral activity of *M. oleifera* compounds against SARS-CoV-2. The compound with the most negative binding activity to target proteins is predicted to play an essential role. The findings show that rutin had strong molecular interactions with all of the targets that were tested, and isoquercitrin had interactions with three targets, which were spike, M^pro^, and RBD. Thus showing that these compounds can be promising antiviral inhibitors against more than one target of interest in the search for therapeutics against the virus.

The outcomes indicate that five chemicals (luteolin, myricetin, ellagic acid, and rutin) had the worst molecular interactions with the spike protein. The ellagic acid that had the lowest binding affinity index in this study has a number of medical benefits, such as protecting cells from damage, reducing inflammation, and protecting nerves and the liver [53]. According to studies, the compound also has strong anticancer activity [54], as well as other important biological functions like chemoprevention and antiviral activities [55]. It has also been shown to stop mutations and reduce inflammation in bacteria and mammals.

Rutin is a flavonoid phytochemical compound present in a variety of plants with pharmacological properties for the prevention of various diseases. Its bioactive effects include antiviral [56], anti-asthma [57], antimicrobial [58], anti-inflammatory [59], and antioxidant activities [60]. On the other hand, myricetin is a compound widely found in various human foods and beverages and is known for its diverse pharmacological properties, including antioxidant, anti-inflammatory, and anticancer effects [61], being antitumor [62], antibacterial [63], and antiviral [64,65].

Quercetin is also a flavonoid found in several plants and is considered a potent natural compound with biological properties. In silico and in vitro studies show that this compound has many health benefits, including fighting cancer, reducing inflammation, lowering blood pressure, preventing diabetes, reducing allergies, lowering cholesterol, preventing blood clots, and boosting mood [66,67,68]. Thanks to its pharmacological properties, the luteolin compound is found in many plants that people eat and that are used in traditional medicine to treat a wide range of illnesses. The compound has many biological effects [69], such as anti-inflammatory [70], antioxidant [71], anticancer [72], antibacterial [73], and antiviral [74] properties.

Many virtual screening studies of natural compounds were conducted to evaluate their antiviral activity against SARS-CoV-2. This research covers a wide range of natural compounds, including polyphenols and flavonoids, and reveals the antiviral potential of these substances. They demonstrated the ability to inhibit the main proteases of the virus, which positions them as promising therapeutic agents for the treatment of COVID-19 [75]. According to Aini et al., (2022) [76], in their in silico study on bioactive compounds with potential against SARS-CoV-2, the compounds ellagic acid and myricetin were identified as candidates that meet Lipinski’s criteria, suggesting their viability as anti-SARS-CoV-2 agents. Our results are similar regarding the antiviral potential of these bioactive compounds. However, it is essential to highlight that additional investigations are necessary to substantiate and validate these results.

In silico studies conducted by Mawaddani et al., (2022) [77] on *M. oleifera* also suggest that this herb might be a potential candidate against SARS-CoV-2 infection. In this study, quercetin was believed to act against SARS-CoV-2, possibly through inhibiting viral entry and binding to the active sites of both the main protease (M^pro^) and RNA-dependent RNA polymerase (RdRp) of it, demonstrating quercetin as a potential drug candidate against SARS-CoV-2. Inhibition of targets in the SARS-CoV-2 life cycle plays a crucial role in blocking essential processes required for virus life cycle progression, resulting in infection control. The main access route of SARS-CoV-2 to cells occurs through the interaction of the spike (S) protein with the ACE2 receptor [78], which is an essential component of the SARS-CoV-2 nucleocapsid and plays a fundamental role in recognizing cellular receptors. Thus, the interaction between protein S and ACE2 facilitates the cell membrane fusion process, allowing the virus to enter cells [79,80]. Compounds with the ability to inhibit the interaction between the S protein and ACE2 can prevent the fusion process, resulting in blocking virus entry.

It’s important to note that only a few of the natural products that were tested against the receptor binding domain (RBD) of SARS-CoV-2 were able to stop the spike protein from interacting with its receptor ACE2. Some of these molecules, like nimbin, curcumin, withaferin A, mangiferin, piperine, thebaine, andrographolide, and berberine, were found to be good at stopping this process [81].

The term “major protease”, or M^pro^, is used due to its critical function in coronavirus gene expression and replicase processing [33]. Based on the results obtained, the compounds b-amyrin and stigmasta-5,22-dien-3-ol demonstrated potential as main protease inhibitors (M^pro^) of SARS-CoV-2. From the ADMET predictions and the assessment of biological activity, it is possible to safely infer that these compounds have the ability to exhibit antiviral activity [34]. Several studies are focused on the search for SARS-CoV-2 M^pro^ inhibitors, as inhibition of this enzyme has the potential to block viral replication, making it an attractive target for the development of antiviral drugs against SARS-CoV-2.

The drug repositioning strategy, which is a standard method used in pharmaceutical development research, tries to find new medical uses for drugs that have already been approved or are still in the testing phase [82]. Several studies were conducted with the purpose of exploring the application of medicines already available on the pharmaceutical market as an alternative approach to combating SARS-CoV-2 [83]. The drugs that stand out are ombitasvir and ledispavir [84], as well as chloroquine, atazanavir, and oseltamivir [85]. Other drugs that stand out are baricitinib, molnupiravir, remdesivir, and paxlovid [86].

The drugs baricitinib, molnupiravir, remdesivir, and paxlovid were tested in clinical trials to see how well they could fight COVID-19 [87]. Based on the results, Anvisa gave these drugs the green light to be used to fight the disease [50]. When the molecular affinity between these drugs and the SARS-CoV-2 targets was looked at, it was seen that natural compounds from the *M. oleifera* plant had lower binding energy values than these drugs. This indicates a remarkable inhibitory activity of the natural compounds towards the tested targets.

Oral administration and high solubility are important parts of drug discovery plans for full absorption. Low solubility, on the other hand, limits absorption in the digestive tract. Predictions of pharmacokinetic and toxicity parameters (ADME-TOX) revealed that the majority of compounds demonstrated reasonable water solubility. All molecules proved to be permeable to the skin. The assessment of skin permeability is essential to understanding the ability of a molecule to cross the layers of the epidermis and dermis, which is relevant in the development of transdermal drug delivery systems [88].

As a way to test how well water-soluble drugs dissolve and pass through cells, Caco-2 cells were created. These cells are grown in transwell cell culture plates and come from a type of human colon cancer. This made it possible to predict how quickly they would absorb after oral administration [89]. Notably, the compounds apigenin and ergosterol had a lot of permeability in Caco-2 cells, and they also had a lot of permeability in the mouth.

One of the main parameters for evaluating new drug candidates is the analysis of human intestinal absorption (AIH), in which molecules with absorption values between 70% and 100% indicate good intestinal absorption [90,91]. The intestine generally represents the main site of absorption for orally administered medications, and most of the molecules analyzed showed potential for intestinal absorption. Among these molecules, ellagic acid, quercetin, luteolin, isorhamnetin, kaempferol, lutein, catechin, apigenin, epicatechin, brassicasterol, stigmasterol, and ergosterol stand out.

The steady-state volume of distribution (VDss) is a theoretical parameter that estimates how much of a drug needs to be spread out evenly in order to reach the same concentration as blood plasma [34]. It was observed that all analyzed compounds have a greater probability of distribution in plasma compared to tissues. In the context of distribution parameters, the BBB plays a fundamental role in protecting the brain against harmful substances. The ability of a drug to cross this barrier is a critical criterion to be considered to reduce side effects, toxicities, or improve the effectiveness of pharmacological treatments in the brain [92]. Our results suggest that only the compounds brassicasterol, stigmasterol, and ergosterol have the ability to cross the BBB. However, it is important to note that these compounds also demonstrated inadequate water solubility and inhibited both P-glycoprotein I and P-glycoprotein II. 

The P-glycoprotein (P-gp), responsible for the absorption, distribution, metabolism, and excretion of several drugs [93], is an ATPase transmembrane that plays a significant role as a defense mechanism against harmful agents, promoting the pumping of toxins and xenobiotic substances out of cells. This P-gp plays a vital role as a biological barrier by expelling toxins and xenobiotics out of cells, thus protecting cell integrity [88,93]. These results indicate that although these compounds have the ability to access the brain, they may present additional challenges in terms of bioavailability and interactions with transporter proteins.

A protein called OCT2 (organic cation transporter 2) is very important for the absorption, distribution, and renal clearance of many different drugs. Assessment of a drug candidate’s ability to be transported by OCT2 provides valuable information not only about its elimination but also about possible contraindications [92]. Our in silico analyses revealed that none of the evaluated compounds are substrates of human OCT2. This result is relevant in the context of the excretion of cationic molecules and suggests that these compounds may not interact significantly with the transport system mediated by OCT2 in the human body.

The Ames toxicity test is a method used to evaluate the mutagenic potential of a compound using bacteria [41,94]. The results obtained are negative for most compounds. These results indicate that these compounds did not demonstrate toxicity in the test and, therefore, do not have mutagenic or carcinogenic potential. This is an important finding, as it suggests that these compounds can be considered safe in terms of mutagenic toxicity.

In this study, we used in silico approaches to show that compounds from *M. oleifera* might be useful in COVID-19. We used molecular docking assessments and ADME-TOX predictions to evaluate their therapeutic potential against COVID-19. It is important to note that, although our results indicate promising antiviral potential, experimental validation is necessary to confirm the activity of the tested compounds of *M. oleifera* against SARS-CoV-2. The computational predictions provide a valid reason for in vitro and in vivo studies against SARS-CoV-2 of the herb *M. oleifera*.

## 5. Conclusions

Twenty-two compounds of *M. oleifera* showed inhibitory potential against SARS-CoV-2 proteins, which are crucial for virus infection and replication in host cells. Among them, ellagic acid, rutin, myricetin, quercetin, and luteolin were the most promising candidates that showed significant affinity with the S protein of the virus. Specifically, ellagic acid stood out as a promising candidate, demonstrating the best molecular affinity with the spike protein. This compound also demonstrated a better molecular interaction than the standard antiviral drugs approved by ANVISA for SARS-CoV-2. Furthermore, pharmacokinetic evaluations indicate that ellagic acid has satisfactory solubility and low toxicity, which ensures its viability as a therapeutic option for SARS-CoV-2 infection. It is also important to note that ellagic acid showed no evidence of skin sensitization or carcinogenicity in our in silico study. However, it is crucial to carry out experimental validations to consider these compounds as promising candidates for the treatment of COVID-19.

## Figures and Tables

**Table 1:** Coordinates of the active sites of molecular targets.

Receptor	Reference Amino Acid	Coordinates of Grid	Center Grid Box Size
6VXX	Gly548(A)	center_x = 180.306	size_x = 30 size_y = 30 size_z = 30
		center_y = 211.382	
		center_z = 224.580	
1R42	His374(A)	center_x = 51.467	
		center_y = 73.108	
		center_z = 34.037	
6LU7	Gly143(A)	center_x = −8.918	
		center_y = 17.918	
		center_z = 62.905	
Receptor 5	Phe32(B)	center_x = 0.804	
		center_y = −7.902	
		center_z = −5.193	

**Table 2:** Results of the 244 dockings are carried out with the interaction of 61 ligands with ACE2, M^pro^, spike, and Receptor 5 of SARS-CoV-2. ^a^ Binding energy of the best conformation.

Plant Parts	Isolated Phytoconstituents	CID	Molecular Targets				References
			ACE2	M^pro^	Receptor 5 (RBD)	Spike	
			ΔG _bind_^a^ (kcal/mol)				
Flower	Niazirin	129556	−6.5	−6.8	−6.3	−7.3	Barreto et al., 2009 [42]
Sheet	Linalool	6549	−3.9	−4.3	−5.3	−5.3	
	Geraniol	637566	−4.2	−3.8	−4.7	−5.2	
	Thymol	6989	−5.1	−4.7	−6.4	−5.8	
	Spathulenol	92231	−6.1	−5.6	−6.4	−7.1	
Flower	Pentadecanol	12397	−3.8	−3.9	−5.3	−5.0	
Seed	Palmitic acid	985	−3.9	−4.3	−5.6	−4.9	Ferreira et al., 2008 [43]
Flower	Quercetin	5280343	−7.4	−7.5	−7.0	−9.0	
	Kaempferol	5280863	−6.9	−7.8	−6.9	−8.7	
Seed	Oleic acid	445639	−4.2	−4.2	−5.2	−5.8	
Sheet	Isoquercetrin	5480505	−7.8	−8.9	−8.0	−8.6	Bicas et al., 2019 [44]
	Chlorogenic Acid	1794427	−7.3	−7.6	−7.3	−8.7	
	Lutein	5281243	−7.5	−6.6	−8.7	−7.8	
	Rutin	5280805	−8.2	−8.8	−8.0	−9.1	
Seed	Lauric acid	3893	−3.9	−4.1	−4.9	−4.9	Ozcan, 2020 [45]
	Myristic acid	11005	−4.0	−4.2	−4.9	−4.8	
	Linolenic acid	5280934	−4.6	−4.6	−6.4	−5.8	
	Brassicasterol	5281327	−7.5	−7.0	−7.7	−8.0	
	Campesterol	173183	−7.2	−6.8	−6.9	−7.8	
	Campestanol	119394	−6.9	−6.9	−7.0	−7.9	
	Stigmasterol	5280794	−7.3	−7.0	−7.4	−8.0	
	Ergosterol	444679	−7.6	−7.3	−7.3	−8.0	
	B-sitosterol	222284	−7.0	−6.8	−7.2	−7.9	
	Clerosterol	5283638	−6.7	−6.3	−7.2	−7.6	
	Stigmastanol	241572	−6.5	−6.8	−7.1	−7.9	
Sheet	Zeatin	449093	−5.9	−5.5	−5.9	−6.4	Ahmadu et al., 2020 [46]
	Myricetin	5281672	−7.4	−7.4	−7.3	−9.1	
	Niazin	4472	−7.3	−6.9	−7.3	−7.6	
	2-Furancarboxaldehyde	7362	−3.7	−4.1	−4.2	−4.1	
	Malonic acid	867	−3.7	−4.4	−4.4	−4.3	
	Phenylvaleric acid	16757	−5.1	−5.0	−5.5	−5.8	
	Caffeic acid	689,043	−5.7	−5.7	−6.2	−7.2	
	Quinic acid	6508	−5.2	−5.5	−6.0	−6.5	
Sheet	Ellagic acid	5281855	−7.3	−7.5	−7.5	−9.3	Kou et al., 2018 [47]
	Ferulic acid	445858	−5.3	−5.5	−5.8	−7.0	
	Epicatechin	72276	−6.8	−7.1	−6.3	−8.4	
	Catechin	9064	−7.0	−7.2	−6.6	−8.6	
Leaf and Seed	Glucomoringin	162639104	−7.2	−7.9	−7.9	−8.5	Anzano et al., 2022 [48]
	Trigonelline	5570	−4.5	−4.4	−5.2	−5.1	
Sheet	Isorhamnetin	5281654	−7.0	−7.2	−6.9	−8.8	Bezerra, 2020 [49]
	Cysteine	5862	−3.4	−3.7	−3.9	−4.1	
	Methionine	6137	−3.8	−4.0	−4.2	−4.5	
	Tryptophan	6305	−5.8	−5.8	−5.7	−6.6	
	Lysine	5962	−3.7	−4.3	−4.5	−5.1	
	Serine	5951	−3.6	−4.3	−4.5	−4.3	
	Proline	145742	−4.0	−4.6	−4.2	−4.8	
	Glutamic acid	33032	−4.2	−4.8	−4.8	−5.4	
	Glycine	750	−3.8	−3.6	−3.6	−3.8	
	Arginine	6322	−4.8	−4.8	−5.6	−5.8	
	Histidine	6274	−4.5	−5.1	−5.3	−5.8	
	Valine	1182	−3.8	−4.2	−4.2	−4.8	
	Leucine	6106	−4.2	−4.1	−4.3	−4.9	
	Isoleucine	6306	−3.8	−4.2	−4.4	−5.0	
	Threonine	6288	−3.9	−4.3	−4.6	−4.7	
	Alanine	602	−4.0	−3.8	−4.1	−4.1	
	Aspartic acid	5960	−4.0	−4.7	−4.9	−5.2	
	2,2-Dimethyl-1-pentanol	16911	−3.5	−3.8	−4.3	−4.3	
	3,4-Dimethyl-2-Hexanol	140547	−3.7	−3.8	−4.2	−4.6	
	4-Methyl-2,3-hexadien-1-ol	566111	−3.6	−3.7	−4.6	−4.4	
	Luteolin	5280445	−7.1	−7.5	−7.2	−9.0	
	Apigenin	5280443	−6.7	−7.7	−7.0	−8.6	

**Table 3:** Molecular affinity parameters of the chemical constituents of *Moringa oleifera* with ACE2, spike, M^pro^, and RBD of SARS-CoV-2.

Complex (Protein Binding)	ΔG _bind_^a^ (kcal/mol)	Amino Acids That Interacted by Hydrogen Bonding	Amino Acids That Interacted by Hydrophobic Bonding
Elagic acid/spike	−9.3	Asn978, Leu977, Arg1000, Tyr741, Met740, Thr549	Phe541, Val976, Gly744, Gly548
Rutin/spike	−9.1	Ser967, Ser968, Leu754, Ser50, His49, Thr51, Gln52, Asn969, Ser975, Asp568, Ile569	Asp571, Gly757, Arg567, Gln755, His519
Myricitin/spike	−9.1	Thr549, Gly744, Arg1000, Tyr741	Phe541, Gly548, Leu977, Leu966, Asn856, Met740, Thr572, Thr573, Pro589, Ile587
Quercetin/spike	−9.0	Thr549, Gly744, Tyr741, Arg1000, Ile742, Met740	Phe541, Gly548, Ile587, Thr572, Pro589, Thr573
Luteolin/spike	−9.0	Met740, Phe855, Thr573, Arg1000, Tyr741	Gly744, Asn856, Gly548, Thr547, Leu546, Asn978, Val976, Thr572, Leu966
Isoquercetrin/M^pro^	−8.9	Phe140, Leu141, Ser144, Thr26, Asp187, Tyr54, Asn142, Glu166	Cys145, Gly143, Leu27, His41, Met49, Arg188, Met165, Gln189, His163, His164
Rutin/M^pro^	−8.8	Leu141, Phe140, Asn142, Gly143, His41, Thr26, Thr190, Glu166, Ser144, His163	Leu27, Thr25, Cys145, Arg188, Met165, Gln189, His164
Isorhamnetin/spike	−8.8	Arg1000, tyr741, Gly744, Thr549	Ile587, Thr572, Pro589, Thr573, Ser975, Leu977, Val976, Met740, Leu966, Phe541, Gly548
Kaempferol/spike	−8.7	Tyr741, Arg1000, Leu977, Thr573, Phe855	Leu966, Val976, Leu546, Thr547, Asn978, Thr572, Asn856, Met740, Gly744
Chlorogenic acid/spike	−8.7	Tyr741, Gly744, Asn978, Thr573, Asp568	Ile587, Lys854, Pro589, Phe855, Leu966, Arg1000, Leu977, Thr572, Asp574,
Lutein/RBD	−8.7	Ser77	Phe72, Phe40, Glu37, Arg393, Lys353, Gly352, Phe356, Met383, Ala386, Gly354, Tyr505, Phe390, Phe32, Leu391, Leu100
Isoquercetrin/spike	−8.6	Arg567, Asp568, Asp571, Gly757, Ser50, His49	Val47, Ile569, Arg44, Ser967, Ser968, Leu754, Gln755, Lys964
Catechin/spike	−8.6	Thr549, Arg1000, Ile742, Tyr741, Asn856	Leu546, Thr573, Thr547, Asn978, Gly744, Leu966, Phe541, Gly548
Apigenin/spike	−8.6	Gly744, Tyr741, Ile742	Thr573, Asn978, Val976, Leu977, Arg1000, Thr572, Ile587
Glucomoringin/spike	−8.5	Ser974, Ser975, Asp571, Thr430, Arg983, Ser514, Ile973, Asn969	His519, Arg567, Val976, Asp979, Phe429, Pro426, Phe515, Phe464, Tyr200, Leu518, Glu516, Leu517,
Epicatechin/spike	−8.4	Thr547, Arg1000, Tyr741, Met740, Asp745	Gly548, Asn978, Thr572, Ile742, Gly744, Asn856, Thr549, Pro589, Phe541, Ile587
Rutin/ACE2	−8.2	Glu398, Tyr385, Asp382, Asp350, Ala348, Ser47, Ser44	Arg514, Asn394, Thr347, Trp349, Phe40, His401, Glu402
Isoquercitrin/RBD	−8.0	Arg393, Glu37, Tyr505, Asp405, Lys417, Asp30, Asn33, His34, Arg403, Tyr453	Gln409, Ile418, Gly416, Leu455
Rutin/RBD	−8.0	Glu37, Tyr453, His34, Ala386, Arg393, Gln388, Asp405, Arg403	Glu406, Lys417, Ile418, Gln409, Asn33, Leu455, Ala387, Tyr505
Brassicasterol/spike	−8.0		Ile973, Ser974, Arg983, Leu518, Thr430, Glu516, Phe515, Tyr200, Leu517
Stigmasterol/spike	−8.0	Asp571	Val976, Asp979, His519, Leu517, Glu516, Ser514, Pro426, Phe429, Phe515, Phe464, Thr430, Leu518, Ile973, Ser974, Arg567
Ergosterol/spike	−8.0	Ser974	Tyr200, Glu516, Leu518, Ile973, Arg983, Leu517, Phe515, Thr430

**Table 4:** Molecular affinity parameters of baricitinib, molnupiravir, paxlovid, and remdesivir drugs with ACE2, M^pro^, ACE2/S complex, and spike proteins of SARS-CoV-2.

Drugs	CID	ACE2 Protein	M^pro^ Protein	RBD	Spike Protein
Baricitinib	44,205,240	−6.8	−7.9	−7.8	−8.0
Molnupiravir	145,996,610	−7.2	−6.7	−6.8	−7.9
Paxlovid (Nirmatrelvir + Ritonavir)	155,903,259	−7.1	−7.6	−7.0	−7.3
Remdesivir	121,304,016	−7.3	−7.9	−7.6	−7.5

**Table 5:** Absorption and distribution properties of *Moringa oleifera* compounds with the best molecular interaction energies.

Compounds	Absorption						Distribution	
	Solubility in Water (log mol/L)	P _Caco2_ (Log Papp at 10^−6^ cm/s)	AIH%	Skin Permeability (log Kp)	P-glycoprotein I Inhibitor	P-glycoprotein II Inhibitor	VDss (huma) (log L/kg)	PBH (BB)
Apigenin	−3.178	1.076	91.856	−2.736	No	No	−0.105	−0.951
Brassicasterol	−6.635	1.209	94.138	−2.798	Yes	Yes	0.232	0.767
Catechin	−3.024	−0.41	72.539	−2.735	No	No	0.589	−1.278
Chlorogenic acid	−2.823	−0.607	18.192	−2.735	No	No	−1.359	−1.737
Ellagic acid	−3.181	0.371	73.933	−2.735	No	No	0.442	−1.426
Epicatechin	−3.024	−0.41	72.539	−2.735	No	No	0.589	−1.278
Ergosterol	−6.612	1.21	94.285	−2.799	Yes	Yes	0.231	0.77
Glucomoringin	−2.901	−0.726	0	−2.735	No	No	−0.598	−2.303
Isoquercitrin	−3.028	−0.755	38.939	−2.735	No	No	−0.287	−2.417
Isorhamnetin	−3.551	0.497	79.101	−2.735	No	No	0.399	−1.283
Kaempferol	−3.332	0.627	81.862	−2.735	No	No	0.078	−1.143
Lutein	−6.838	1.284	88.333	−2.749	No	Yes	−0.29	−0.238
Luteolin	−3.173	0.762	81.082	−2.735	No	No	0.071	−1.199
Myricetin	−2.941	−0.649	65.116	−2.735	No	No	0.209	−1.739
Quercetin	−2.982	0.694	74.84	−2.735	No	No	0.31	−1.377
Rutin	−2.909	−0.662	25.454	−2.735	No	No	−0.155	−2.556
Stigmasterol	−6.671	1.21	94.73	−2.781	Yes	Yes	0.176	0.79

Note: PCaco-2: permeability of Caco-2 cells; AIH: human intestinal absorption potential; P-skin: skin permeability; IGp -P: P-glycoprotein inhibitor; VDss: volume of distribution at steady state; and PBH: blood–brain barrier permeability.

**Table 6:** Metabolism and excretion properties of *Moringa oleifera* compounds with better molecular interaction energies.

Compounds	Metabolism							Excretion
	CYP2D6 Substrate	CYP3A4 Substrate	CYP1A2 Inhibitor	CYP2C19 Inhibitor	CYP2C9 Inhibitor	CYP2D6 Inhibitor	CYP3A4 Inhibitor	OCT2 Renal Substrate
Apigenin	No	No	Yes	Yes	Yes	No	No	No
Brassicasterol	No	Yes	No	No	No	No	No	No
Catechin	No	No	No	No	No	No	No	No
Chlorogenic acid	No	No	No	No	No	No	No	No
Ellagic acid	No	No	Yes	No	No	No	No	No
Epicatechin	No	No	No	No	No	No	No	No
Ergosterol	No	Yes	No	No	No	No	No	No
Glucomoringin	No	No	No	No	No	No	No	No
Isoquercitrin	No	No	No	No	No	No	No	No
Isorhamnetin	No	No	Yes	No	No	No	No	No
Kaempferol	No	No	Yes	No	No	No	No	No
Lutein	No	Yes	No	No	No	No	No	No
Luteolin	No	No	Yes	No	No	No	No	No
Myricetin	No	No	Yes	No	No	No	No	No
Quercetin	No	No	Yes	No	No	No	No	No
Rutin	No	No	No	No	No	No	No	No
Stigmasterol	No	Yes	No	No	No	No	No	No

**Table 7:** Toxicity properties of *Moringa oleifera* compounds with the best molecular interaction energies.

Compounds	Toxicity							
	AMES Toxicity	DMT (Human) (Log mg/kg/day)	hERG I Inhibitor	hERG II Inhibitor	TAO (Rats) (LD50) (mol/kg)	TAO (Rats) (LOAEL) (log mg/kg.bw/Day)	Hepatotoxicity	S-Skin
Apigenin	No	0.931	No	Yes	2.376	1.461	No	No
Brassicasterol	No	−0.725	No	Yes	2.286	0.825	No	No
Catechin	Yes	0.516	No	No	2011	2.919	No	No
Chlorogenic acid	No	1.327	No	No	2.229	3.618	No	No
Ellagic acid	No	0.806	No	No	2.45	2.555	No	No
Epicatechin	Yes	0.516	No	No	2011	2.919	No	No
Ergosterol	No	−0.731	No	Yes	2.28	0.824	No	No
Glucomoringin	No	0.416	No	No	2.473	4.372	No	No
Isoquercitrin	Yes	0.814	No	Yes	2.812	3.382	No	No
Isorhamnetin	No	0.882	No	No	2.358	2.804	No	No
Kaempferol	No	1.020	No	No	2.228	2.662	Yes	No
Lutein	No	−1.237	No	Yes	2.590	2.543	No	No
Luteolin	No	0.975	No	No	2.450	1833	No	No
Myricetin	Yes	0.621	No	No	2.645	3.475	No	No
Quercetin	Yes	0.954	No	No	2.308	3.134	No	No
Rutin	Yes	0.550	No	Yes	2.523	4.415	No	No
Stigmasterol	No	−0.639	No	Yes	2.345	0.802	No	No

Note: T.AMES: AMES toxicity; DMT: maximum tolerated dose in humans; TAO: acute oral toxicity in rat; OCT: chronic oral toxicity in rats; and S-skin: skin sensitization.

## Data Availability

The datasets analyzed during the current study are available from the corresponding author on reasonable request.

## References

[1] Wu D., Wu T., Liu Q., Yang Z. (2020). The SARS-CoV-2 outbreak: What we know. Int. J. Infect. Dis..

[2] Zhou P., Yang X.-L., Wang X.-G., Hu B., Zhang L., Zhang W., Si H.-R., Zhu Y., Li B., Huang C.-L. (2020). A pneumonia outbreak associated with a new coronavirus of probable bat origin. Nature.

[3] Ahmad I., Beg A.Z. (2001). Antimicrobial and phytochemical studies on 45 Indian medicinal plants against multi-drug resistant human pathogens. J. Ethnopharmacol..

[4] Patra B., Schluttenhofer C., Wu Y., Pattanaik S., Yuan L. (2013). Transcriptional regulation of secondary metabolite biosynthesis in plants. Biochim. Biophys. Acta BBA Gene Regul. Mech..

[5] Anwar F., Latif S., Ashraf M., Gilani A.H. (2007). *Moringa oleifera*: A food plant with multiple medicinal uses. Phytother. Res..

[6] Gassenschmidt U., Jany K.D., Bernhard T., Niebergall H. (1995). Isolation and characterization of a flocculating protein from *Moringa oleifera* Lam. Biochem. Biophys. Acta BBA Gen. Subj..

[7] Matos F.J.A. (2002). Living Pharmacies: A System for Using Medicinal Plants Designed for Small Communities.

[8] Bezerra A.M.E., Momenté V.G., Medeiros Filho S. (2004). Seed germination and seedling development of moringa (*Moringa oleifera* Lam.) as a function of seed weight and substrate type. Hortic Bras..

[9] Makkar H., Becker K. (1996). Nutrional value and antinutritional components of whole and ethanol extracted *Moringa oleifera* leaves. Anim. Feed. Sci. Technol..

[10] Mehta K., Balaraman R., Amin A., Bafna P., Gulati O. (2003). Effect of fruits of *Moringa oleifera* on the lipid profile of normal and hypercholesterolaemic rabbits. J. Ethnopharmacol..

[11] Jaiswal D., Rai P.K., Kumar A., Mehta S., Watal G. (2009). Effect of *Moringa oleifera* Lam. leaves aqueous extract therapy on hyperglycemic rats. J. Ethnopharmacol..

[12] Waterman C., Cheng D.M., Rojas-Silva P., Poulev A., Dreifus J., Lila M.A., Raskin I. (2014). Stable, water extractable isothiocyanates from *Moringa oleifera* leaves attenuate inflammation in vitro. Phytochemistry.

[13] Ramachandran C., Peter K.V., Gopalakrishnan P.K. (1980). Drumstick (*Moringa oleifera*): A multipurpose Indian vegetable. Econ. Bot..

[14] Gasteiger J. (2006). The central role of chemoinformatics. Chemom. Intell. Lab. Syst..

[15] Oprea T.I., Matter H. (2004). Integrating virtual screening in lead discovery. Curr. Opin. Chem. Biol..

[16] Rocha J.A., Rego N.C.S., Carvalho B.T.S., Silva F.I., Sousa J.A., Ramos R.M., Passos I.N.G., de Moraes J., Leite J.R.S.A., Lima F.C.A. (2018). Computational quantum chemistry, molecular docking, and ADMET predictions of imidazole alkaloids of *Pilocarpus microphyllus* with schistosomicidal properties. PLoS ONE.

[17] Abe R., Ohtani K. (2013). An ethnobotanical study of medicinal plants and traditional therapies on Batan Island, the Philippines. J. Ethnopharmacol..

[18] Leone A., Spada A., Battezzati A., Schiraldi A., Aristil J., Bertoli S. (2015). Cultivation, Genetics, Ethnopharmacology, Phytochemistry and Pharmacology of *Moringa oleifera* Leaves: An Overview. Int. J. Mol. Sci..

[19] Razis A.F.A., Ibrahim M.D., Kntayya S.B. (2014). Health Benefits of *Moringa oleifera*. Asian Pac. J. Cancer Prev..

[20] Padayachee B., Baijnath H. (2020). An updated comprehensive review of the medicinal, phytochemical and pharmacological properties of *Moringa oleifera*. S. Afr. J. Bot..

[21] Muratov E.N., Amaro R., Andrade C.H., Brown N., Ekins S., Fourches D., Isayev O., Kozakov D., Medina-Franco J.L., Merz K.M. (2021). A critical overview of computational approaches employed for COVID-19 drug discovery. Chem. Soc. Rev..

[22] Mishra B.B., Tiwari V.K. (2011). Natural products: An evolving role in future drug discovery. Eur. J. Med. Chem..

[23] Mehyar N. (2023). Coronaviruses SARS-CoV, MERS-CoV, and SARS-CoV-2 helicase inhibitors: A systematic review of in vitro studies. J. Virus Erad..

[24] Power H., Wu J., Turville S., Aggarwal A., Valtchev P., Schindeler A., Dehghani F. (2022). Virtual screening and in vitro validation of natural compound inhibitors against SARS-CoV-2 spike protein. Bioorg. Chem..

[25] Guedes I.A., de Magalhães C.S., Dardenne L.E. (2014). Receptor-ligand molecular docking. Biophys. Rev..

[26] Meng X.-Y., Zhang H.-X., Mezei M., Cui M. (2011). Molecular docking: A powerful approach for structure-based drug discovery. Curr. Comput. Aided Drug Des..

[27] Jin Z., Du X., Xu Y., Deng Y., Liu M., Zhao Y., Zhang B., Li X., Zhang L., Peng C. (2020). Structure of Mpro from SARS-CoV-2 and discovery of its inhibitors. Nature.

[28] Berman H.M., Westbrook J., Feng Z., Gilliland G., Bhat T.N., Weissig H., Shindyalov I.N., Bourne P.E. (2000). The Protein Data Bank. Nucleic Acids Res..

[29] Barros R.O., Junior F.L.C.C., Pereira W.S., Oliveira N.M.N., Ramos R.M. (2020). Interaction of Drug Candidates with Various SARS-CoV-2 Receptors: An in Silico Study to Combat COVID-19. J. Proteome Res..

[30] Pettersen E.F., Goddard T.D., Huang C.C., Couch G.S., Greenblatt D.M., Meng E.C., Ferrin T.E. (2004). UCSF Chimera?A visualization system for exploratory research and analysis. J. Comput. Chem..

[31] Trott O., Olson A.J. (2010). AutoDock Vina: Improving the speed and accuracy of docking with a new scoring function, efficient optimization, and multithreading. J. Comput. Chem..

[32] Wallace A.C., Laskowski R.A., Thornton J.M. (1995). Ligplot: A program to generate schematic diagrams of protein-ligand interactions. Protein Eng..

[33] Anand K., Ziebuhr J., Wadhwani P., Mesters J.R., Hilgenfeld R. (2003). Coronavirus main proteinase (3CLpro) structure: Basis for design of anti-SARS drugs. Science.

[34] Kumar S.B., Krishna S., Pradeep S., Mathews D.E., Pattabiraman R., Murahari M., Murthy T.K. (2021). Screening of natural compounds from Cyperus rotundus Linn against SARS-CoV-2 main protease (Mpro): An integrated computational approach. Comput. Biol. Med..

[35] Glaab E., Manoharan G.B., Abankwa D. (2021). Pharmacophore Model for SARS-CoV-2 3CLpro Small-Molecule Inhibitors and In Vitro Experimental Validation of Computationally Screened Inhibitors. J. Chem. Inf. Model.

[36] Tang T., Bidon M., Jaimes J.A., Whittaker G.R., Daniel S. (2020). Coronavirus membrane fusion mechanism offers a potential target for antiviral development. Antivir. Res..

[37] Santos E.S., Silva P.C., Sousa P.S.A., Aquino C.C., Pacheco G., Teixeira L.F.L.S., Araujo A.R., Sousa F.B.M., Barros R.O., Ramo R.M. (2022). Antiviral potential of diminazene aceturate against SARS-CoV-2 proteases using computational and in vitro approaches. Chem. Biol. Interact..

[38] Ho C., Nazarie W.F.W.M., Lee P.-C. (2023). An In Silico Design of Peptides Targeting the S1/S2 Cleavage Site of the SARS-CoV-2 Spike Protein. Viruses.

[39] Lan J., Ge J., Yu J., Shan S., Zhou H., Fan S., Zhang Q., Shi X., Wang Q., Zhang L. (2020). Structure of the SARS-CoV-2 spike receptor-binding domain bound to the ACE2 receptor. Nature.

[40] Pokhrel S., Bouback T.A., Samad A., Nur S.M., Alam R., Abdullah-Al-Mamun M., Nain Z., Imon R.R., Talukder E.K., Tareq M.I. (2021). Spike protein recognizer receptor ACE2 targeted identification of potential natural antiviral drug candidates against SARS-CoV-2. Int. J. Biol. Macromol..

[41] Pires D.E.V., Blundell T.L., Ascher D.B. (2015). pkCSM: Predicting Small-Molecule Pharmacokinetic and Toxicity Properties Using Graph-Based Signatures. J. Med. Chem..

[42] Barreto M.B., De Freitas J.V.B., Silveira E.R., Bezerra A.M.E., Nunes E.P., Gramosa N.V. (2009). Volatile and non-volatile chemical constituents of *Moringa oleifera* Lam., Moringaceae. Braz. J. Pharmacogn..

[43] Ferreira P.M.P., Farias D.F., de Abreu Oliveira J.T., de Fátima Urano Carvalho A. (2008). *Moringa oleifera*: Bioactive compounds and nutritional potential. Rev. Nutr..

[44] Bicas T.C. Effects of Hydroalcoholic Extract of Syzygium Leaves Malaccense and Moringa oleifera under Oxidative Stress in Streptozotocin—Induced Diabetic Rats. 26 August 2019. http://repositorio.utfpr.edu.br:8080/jspui/handle/1/4590.

[45] Özcan M.M. (2020). *Moringa* spp: Composition and bioactive properties. S. Afr. J. Bot..

[46] Ahmadu T., Ahmad K., Ismail S.I., Rashed O., Asib N., Omar D. (2021). Antifungal efficacy of *Moringa oleifera* leaf and seed extracts against Botrytis cinerea causing gray mold disease of tomato (*Solanum lycopersicum* L.). Braz. J. Biol..

[47] Kou X., Li B., Olayanju J.B., Drake J.M., Chen N. (2018). Nutraceutical or Pharmacological Potential of *Moringa oleifera* Lam. Nutrients.

[48] Anzano A., de Falco B., Ammar M., Ricciardelli A., Grauso L., Sabbah M., Capparelli R., Lanzotti V. (2022). Chemical Analysis and Antimicrobial Activity of *Moringa oleifera* Lam. Leaves and Seeds. Molecules.

[49] MSS Calf (2020). Identification of Organic Compounds Present in Ethanolic and Hexanic Extracts of Moringa oleifera LAM Leaves.

[50] Approved Drugs-National Health Surveillance Agency-Anvisa. https://www.gov.br/anvisa/pt-br/assuntos/paf/coronavirus/medicamentos.

[51] Ferreira L.L.G., Andricopulo A.D. (2020). Medicines and treatments for COVID-19. Adv. Stud..

[52] Neto I.F.D.S., Ricardino I.E.F., dos Santos Í.T., de Lima E.V.M., Souza M.N.C., Marques A.E.F., Silva M.R. (2021). A review of the antiviral activity of the Indian Nim and its potential in front of the new coronavirus (SARS-CoV-2). J. Biol. Pharm. Agric. Manag..

[53] García-Niño W.R., Zazueta C. (2015). Ellagic acid: Pharmacological activities and molecular mechanisms involved in liver protection. Pharmacol. Res..

[54] Vattem D., Shetty K. (2005). Biological functionality of ellagic acid: A review. J. Food Biochem..

[55] Xu Y.-M., Deng J.-Z., Ma J., Chen S.-N., Marshall R., Jones S.H., Johnson R.K., Hecht S.M. (2003). DNA damaging activity of ellagic acid derivatives. Bioorg. Med. Chem..

[56] Ibrahim A.K., Youssef A.I., Arafa A.S., Ahmed S.A. (2013). Anti-H5N1 virus flavonoids from Capparis sinaica Veill. Nat. Prod. Res..

[57] Jung C.H., Lee J.Y., Cho C.H., Kim C.J. (2007). Anti-asthmatic action of quercetin and rutin in conscious guinea-pigs challenged with aerosolized ovalbumin. Arch. Pharmacal Res..

[58] Araruna M.K., Brito S.A., Morais-Braga M.F., Santos K.K., Souza T.M., Leite T.R., Costa J.G., Coutinho H.D. (2012). Evaluation of antibiotic & antibiotic modifying activity of pilocarpine & rutin. Indian J. Med. Res..

[59] Umar S., Mishra N.K., Pal K., Sajad M., Neha, Ansari M., Ahmad S., Katiyar C.K., Khan H.A. (2012). Protective effect of rutin in attenuation of collagen-induced arthritis in Wistar rat by inhibiting inflammation and oxidative stress. Indian J. Rheumatol..

[60] Leong C.N.A., Tako M., Hanashiro I., Tamaki H. (2008). Antioxidant flavonoid glycosides from the leaves of *Ficus pumila* L.. Food Chem..

[61] Semwal D.K., Semwal R.B., Combrinck S., Viljoen A. (2016). Myricetin: A dietary molecule with diverse biological activities. Nutrients.

[62] Jiang M., Zhu M., Wang L., Yu S. (2019). Anti-tumor effects and associated molecular mechanisms of myricetin. Biomed. Pharmacother..

[63] Jiang S., Tang X., Chen M., He J., Su S., Liu L., He M., Xue W. (2020). Design, synthesis and antibacterial activities against *Xanthomonas oryzae pv. oryzae*, *Xanthomonas axonopodis pv. Citri* and *Ralstonia solanacearum* of novel myricetin derivatives containing sulfonamide moiety. Pest Manag. Sci..

[64] Ortega J.T., Suárez A.I., Serrano M.L., Baptista J., Pujol F.H., Rangel H.R. (2017). The role of the glycosyl moiety of myricetin derivatives in anti-HIV-1 activity in vitro. AIDS Res. Ther..

[65] Ren R., Yin S., Lai B., Ma L., Wen J., Zhang X., Lai F., Liu S., Li L. (2018). Myricetin antagonizes semen-derived enhancer of viral infection (SEVI) formation and influences its infection-enhancing activity. Retrovirology.

[66] Li Y., Yao J., Han C., Yang J., Chaudhry M.T., Wang S., Liu H., Yin Y. (2016). Quercetin, inflammation and immunity. Nutrients.

[67] Rahaman S.T., Mondal S. (2020). Flavonoids: A vital resource in healthcare and medicine. Pharm. Pharmacol. Int. J..

[68] Wang W., Sun C., Mao L., Ma P., Liu F., Yang J., Gao Y. (2016). The biological activities, chemical stability, metabolism and delivery systems of quercetin: A review. Trends Food Sci. Technol..

[69] López-Lázaro M. (2009). Distribution and biological activities of the flavonoid luteolin. Mini Rev. Med. Chem..

[70] Chen C.Y., Peng W.H., Tsai K.D., Hsu S.L. (2007). Luteolin suppresses inflammation-associated gene expression by blocking NF-κB and AP-1 activation pathway in mouse alveolar macrophages. Life Sci..

[71] Manju V., Balasubramaniyan V., Nalini N. (2005). Rat colonic lipid peroxidation and antioxidant status: The effects of dietary luteolin on 1,2-dimethylhydrazine challenge. Cell. Mol. Biol. Lett..

[72] Han D.-H., Denison M.S., Tachibana H., Yamada K. (2002). Relationship between estrogen receptor-binding and estrogenic activities of environmental estrogens and suppression by flavonoids. Biosci. Biotechnol. Biochem..

[73] Zhu X., Zhang H., Lo R. (2004). Phenolic compounds from the leaf extract of artichoke (*Cynara scolymus* L.) and their antimicrobial activities. J. Agric. Food Chem..

[74] Yi L., Li Z., Yuan K., Qu X., Chen J., Wang G., Zhang H., Luo H., Zhu L., Jiang P. (2004). Small Molecules Blocking the Entry of Severe Acute Respiratory Syndrome Coronavirus into Host Cells. J. Virol..

[75] Da Costa J.A., de Oliveira Lima D., dos Santos Carvalho A.G., Martins J.A., dos Santos M.D.S., de Sousa D.G., da Silva Carvalho G., Barros F.R., Ferreira K.R., de Barros G.M. (2021). Compostos bioativos derivados de matrizes alimentares com potencial terapêutico para a infecção por SARS-CoV-2: Uma revisão de estudos in silico. Res. Soc. Dev..

[76] Aini N.S., Kharisma V.D., Widyananda M.H., Murtadlo A.A.A., Probojati R.T., Turista D.D.R., Tamam M.B., Jakhmola V., Sari D.P., Albari M.T. (2022). In silico screening of bioactive compounds from *Syzygium cumini* L. and *Moringa oleifera* L. against SARS-CoV-2 via tetra inhibitors. Pharmacogn. J..

[77] Mawaddani N., Sutiyanti E., Widyananda M.H., Kharisma V.D., Turista D.D.R., Tamam M.B., Jakhmola V., Syamsurizal, Fajri B.R., Ghifari M.R. (2022). In silico study of entry inhibitor from *Moringa oleifera* bioactive compounds against SARS-CoV-2 infection. Pharmacogn. J..

[78] Letko M., Marzi A., Munster V. (2020). Functional assessment of cell entry and receptor usage for SARS-CoV-2 and other lineage B betacoronaviruses. Nat. Microbiol..

[79] Zumla A., Chan J.F., Azhar E.I., Hui D.S., Yuen K.Y. (2016). Coronaviruses—Drug discovery and therapeutic options. Nat. Rev. Drug Discov..

[80] Sanders J.M., Monogue M.L., Jodlowski T.Z., Cutrell J.B. (2020). Pharmacologic treatments for coronavirus disease 2019 (COVID-19): A review. JAMA.

[81] Maurya V.K., Kumar S., Prasad A.K., Bhatt M.L.B., Saxena S.K. (2020). Structure-based drug designing for potential antiviral activity of selected natural products from Ayurveda against SARS-CoV-2 spike glycoprotein and its cellular receptor. Virusdisease.

[82] Ashburn T.T., Thor K.B. (2004). Drug repositioning: Identifying and developing new uses for existing drugs. Nat. Rev. Drug Discov..

[83] Araújo J.L., Azevedo V.S., Araújo A.D.S., Araújo J.L., Araújo L., Sousa L.A.D., Silva G.T., de Freitas Pinheir W., Santos A.M.A., Cruz G.T. (2020). SARS-CoV-2 or COVID-19: The search for alternative treatment for the new coronavirus. Int. J. Dev. Res..

[84] Bastos R.S., Sousa C.S., Oliveira J.S., da Silva MH V., Lima FD C.A., Rocha J.A. (2020). Prospecção de Proteínas do Novo Coronavírus COVID-2019 e Potencial da Bioinformática na Busca de Novas Drogas Promissoras. Cad. Prospecção.

[85] Araújo J.L., de Sousa L.A., Sousa A.O., Bastos R.S., Santos G.T., Lage M.R., Stoyanov S.R., Passos I.N.G., Azevedo R.B.d., Rocha J.A. (2021). DFT, molecular docking, and ADME/Tox screening investigations of Market-Available drugs against SARS-CoV-2. J. Braz. Chem. Soc..

[86] Dos Santos A.F., Ortiz M.M., Montagner G.E., Schultz J.V., Gomes P., da Silva I.Z., Fagan S.B. (2022). In-Silico study of antivirals and non-antivirals for the treatment of SARS-CoV-2. Discip. Sci. Nat. Tecnol..

[87] Islam T., Hasan M., Rahman M.S., Islam R. (2022). Comparative evaluation of authorized drugs for treating COVID-19 patients. Health Sci. Rep..

[88] Belal A. (2018). Drug likeness, targets, molecular docking and ADMET studies for some indolizine derivatives. Int. J. Pharm. Sci..

[89] De Souza J., Freitas Z.M.F., Storpirtis S. (2007). In vitro models for the determination of drug absorption and a prediction of dissolution/absorption relationships. Braz. J. Pharm. Sci..

[90] Zhao Y.H., Le J., Abraham M.H., Hersey A., Eddershaw P.J., Luscombe C.N., Boutina D., Beck G., Sherborne B., Cooper I. (2001). Evaluation of human intestinal absorption data and subsequent derivation of a quantitative structure-activity relationship (QSAR) with the Abraham descriptors. J. Pharm. Sci..

[91] Yee S. (1997). In vitro permeability across Caco-2 cells (colonic) can predict in vivo (small intestinal) absorption in man—Fact or myth. Pharm. Res..

[92] Chtita S., Belaidi S., Qais F.A., Ouassaf M., AlMogren M.M., Al-Zahrani A.A., Bakhouch M., Belhassan A., Zaki H., Bouachrine M. (2022). Unsymmetrical aromatic disulfides as SARS-CoV-2 Mpro inhibitors: Molecular docking, molecular dynamics, and ADME scoring investigations. J. King Saud. Univ. Sci..

[93] Flores-Holguín N., Frau J., Glossman-Mitnik D. (2021). Computational Pharmacokinetics Report, ADMET Study and Conceptual DFT-Based Estimation of the Chemical Reactivity Properties of Marine Cyclopeptides. ChemistryOpen.

[94] Mortelmans K., Zeiger E. (2000). The Ames *Salmonella*/microsome mutagenicity assay. Mutat. Res. Mol. Mech. Mutagen..

